# Supplementary material for: Hope for OTHERS (Our Tissue Helping Enhance Research & Science): research results from the University of Pittsburgh rapid autopsy program for breast cancer
Source: Breast Cancer Res. 2025 Jun 19;27:111. doi: 10.1186/s13058-025-02014-9 (PMC12180227; doi:10.1186/s13058-025-02014-9)
Supplement: Supplementary file 1 — Supplementary material 1 (Western blots). [file 13058_2025_2014_MOESM1_ESM.pdf]

Hope for Others Manuscript

Western blots

Figure 8B

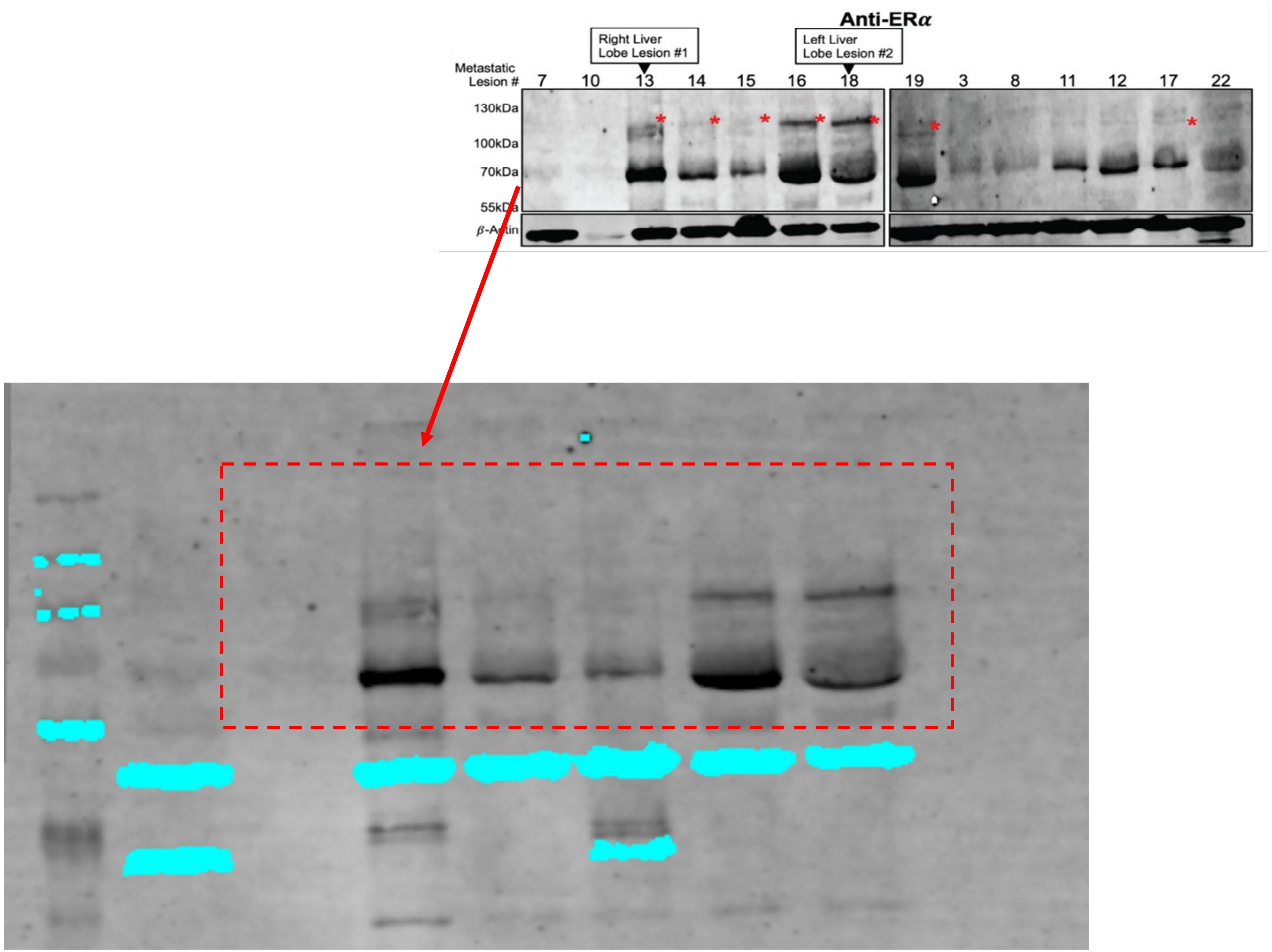
Anti-ERα
Right Liver Lobe Lesion #1
Left Liver Lobe Lesion #2
Metastatic Lesion #
7 10 13 14 15 16 18 19 3 8 11 12 17 22
130kDa
100kDa
70kDa
55kDa
β-Actin

Figure 8B

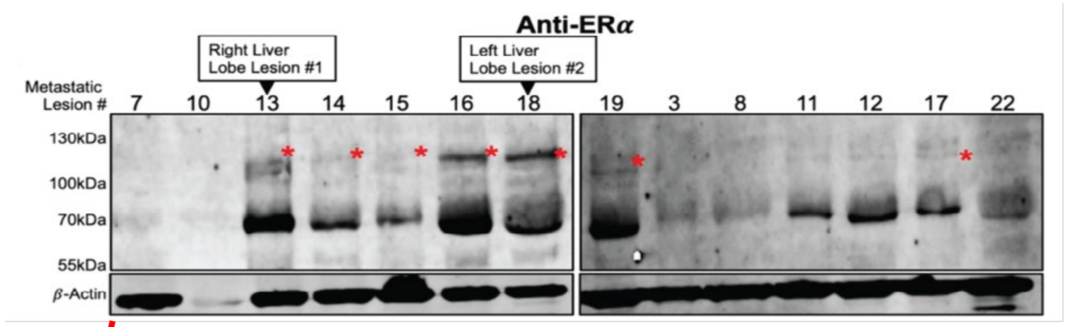

Anti-ERα
Right Liver Lobe Lesion #1
Left Liver Lobe Lesion #2
Metastatic Lesion #
7 10 13 14 15 16 18 19 3 8 11 12 17 22
130kDa
100kDa
70kDa
55kDa
β-Actin

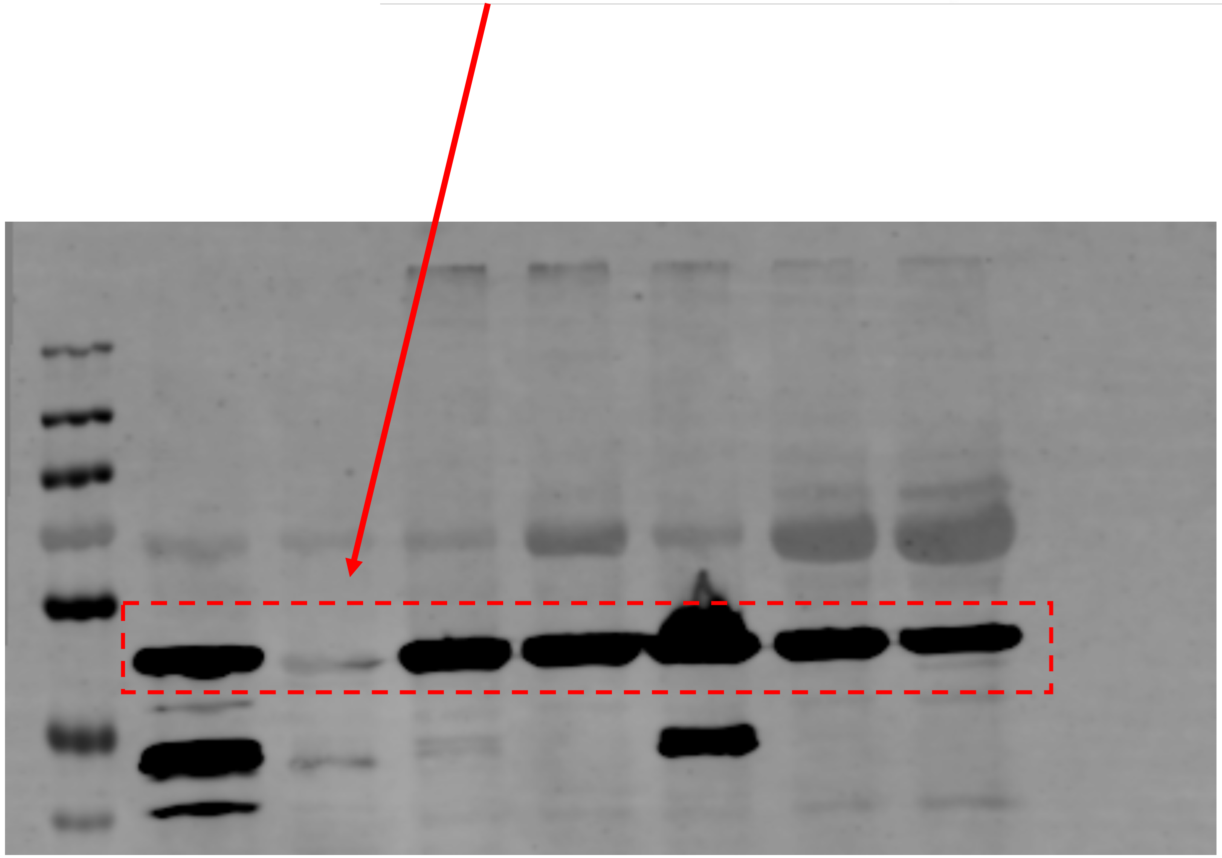

Figure 8B

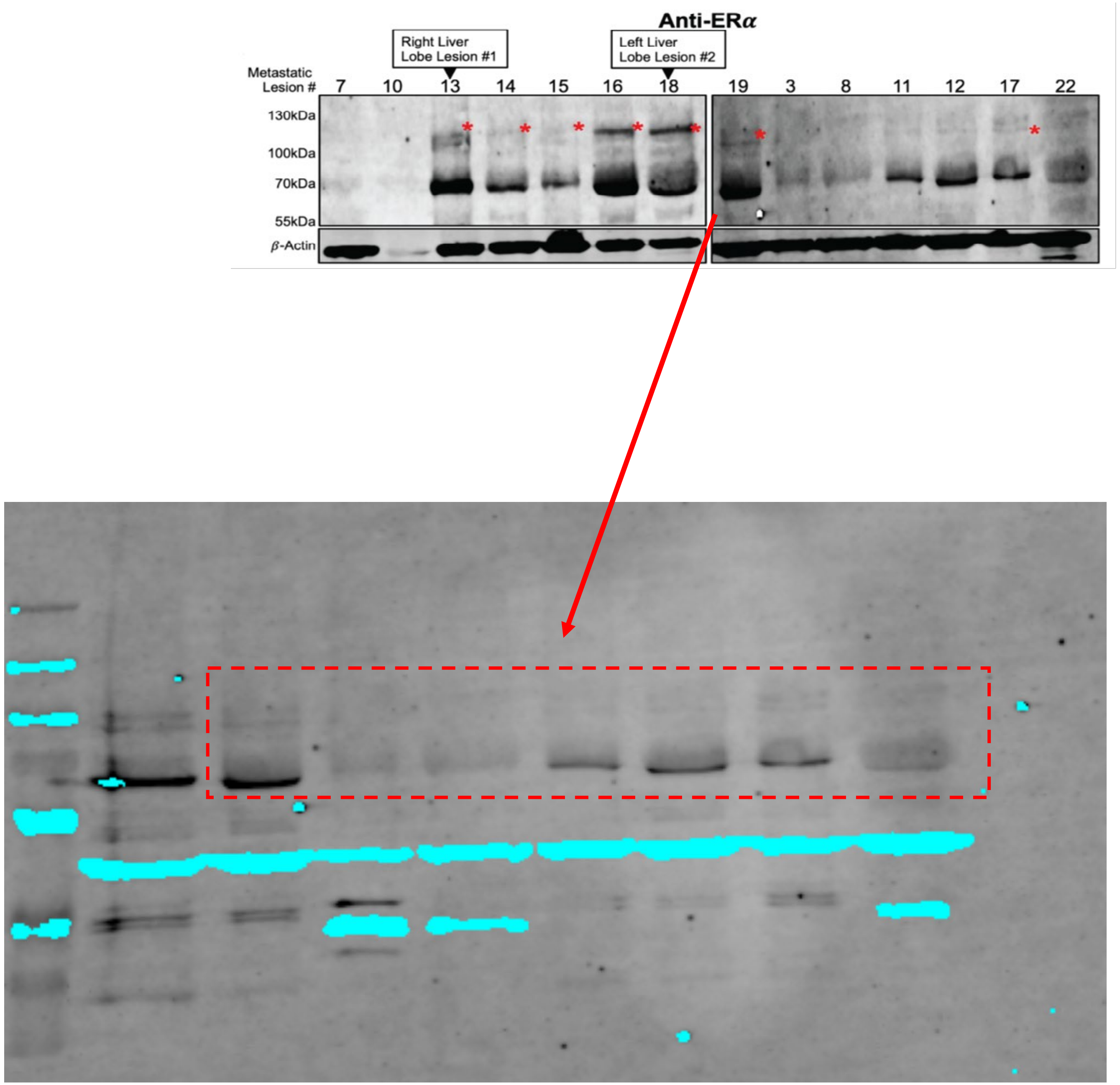

Anti-ERα
Right Liver Lobe Lesion #1
Left Liver Lobe Lesion #2
Metastatic Lesion #
7
10
13
14
15
16
18
19
3
8
11
12
17
22
130kDa
100kDa
70kDa
55kDa
β-Actin

Figure 8B

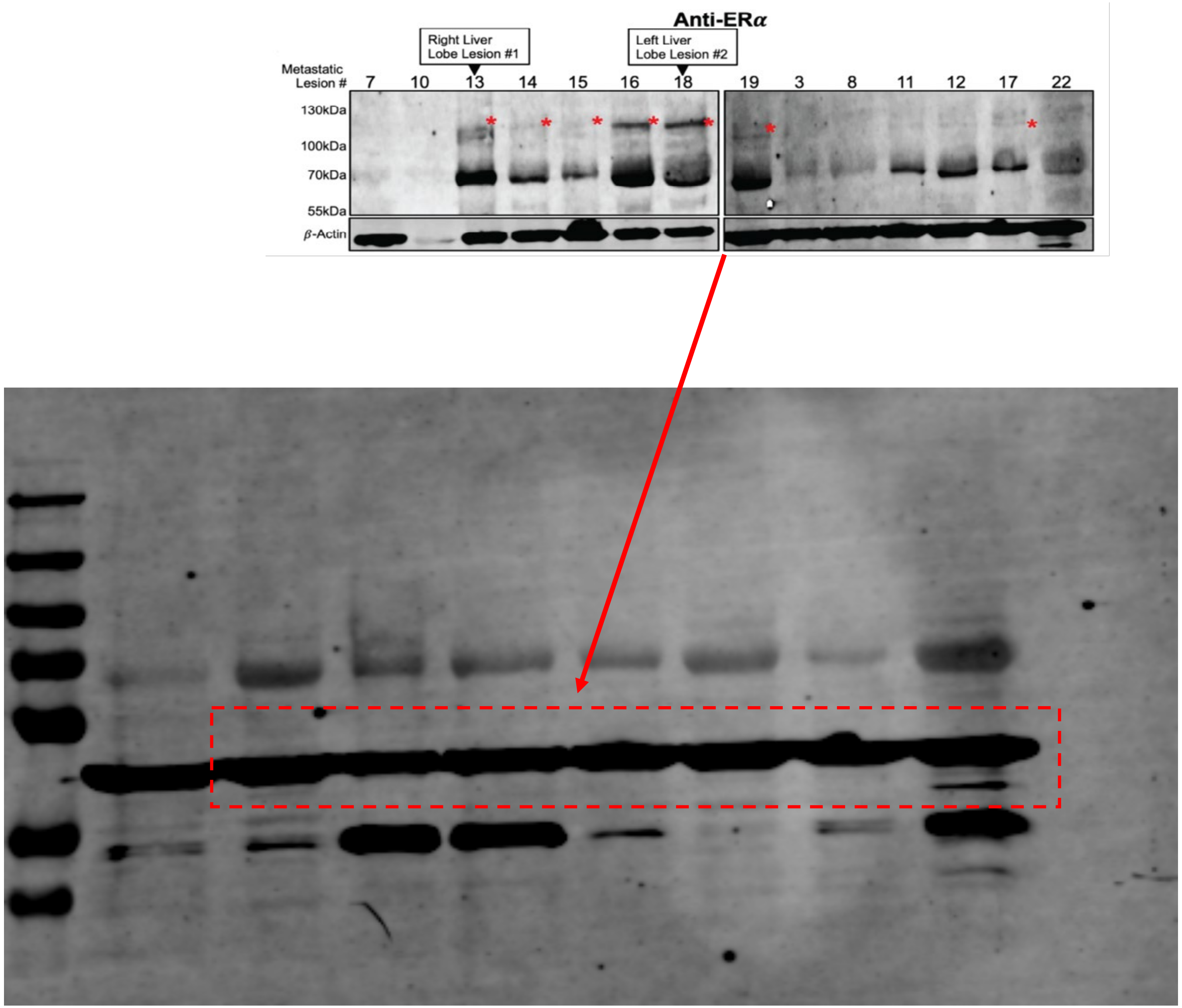
Anti-ERα
Right Liver Lobe Lesion #1
Left Liver Lobe Lesion #2
Metastatic Lesion #
7 10 13 14 15 16 18 19 3 8 11 12 17 22
130kDa
100kDa
70kDa
55kDa
β-Actin

Figure 8D

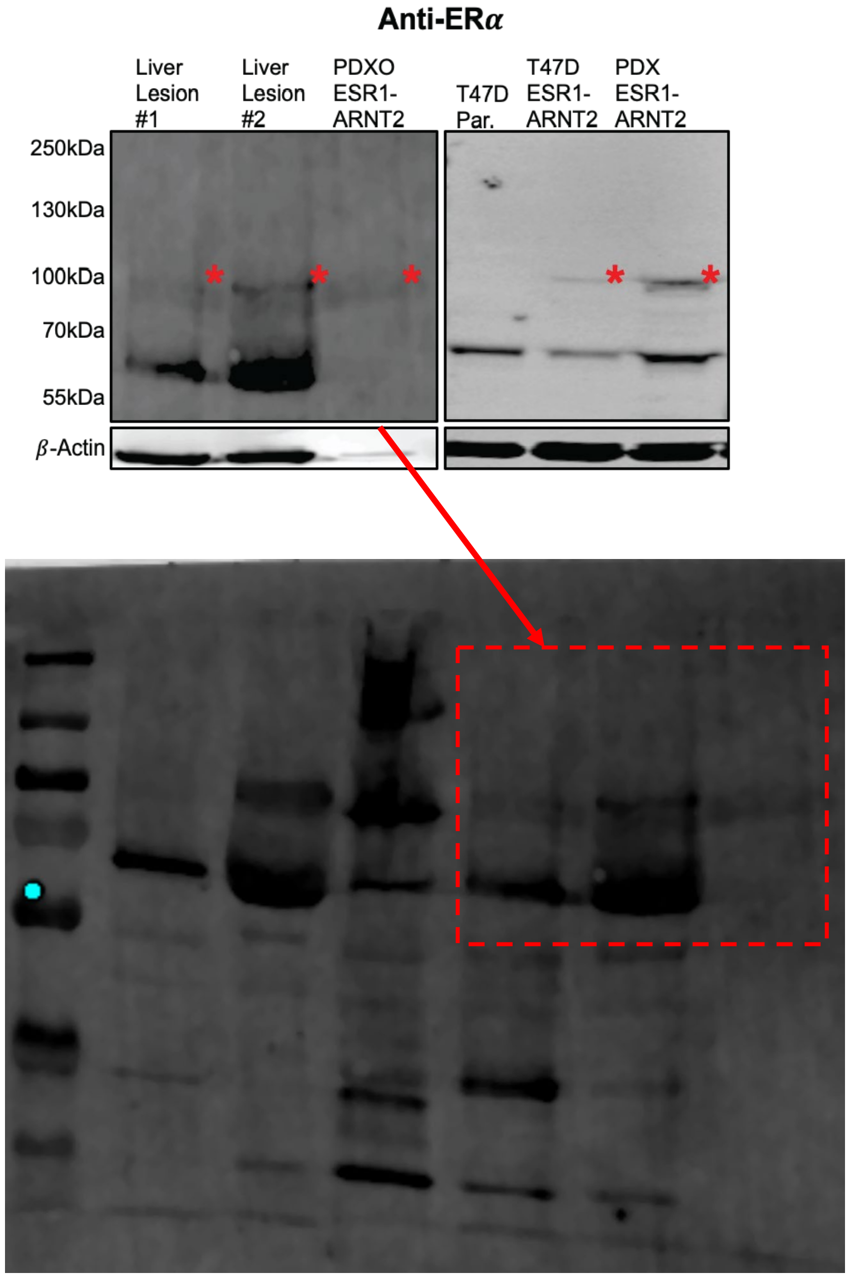
Anti-ERα
Liver Lesion #1
Liver Lesion #2
PDXO ESR1-ARNT2
T47D Par.
T47D ESR1-ARNT2
PDX ESR1-ARNT2
250kDa
130kDa
100kDa
70kDa
55kDa
β-Actin

Figure 8D

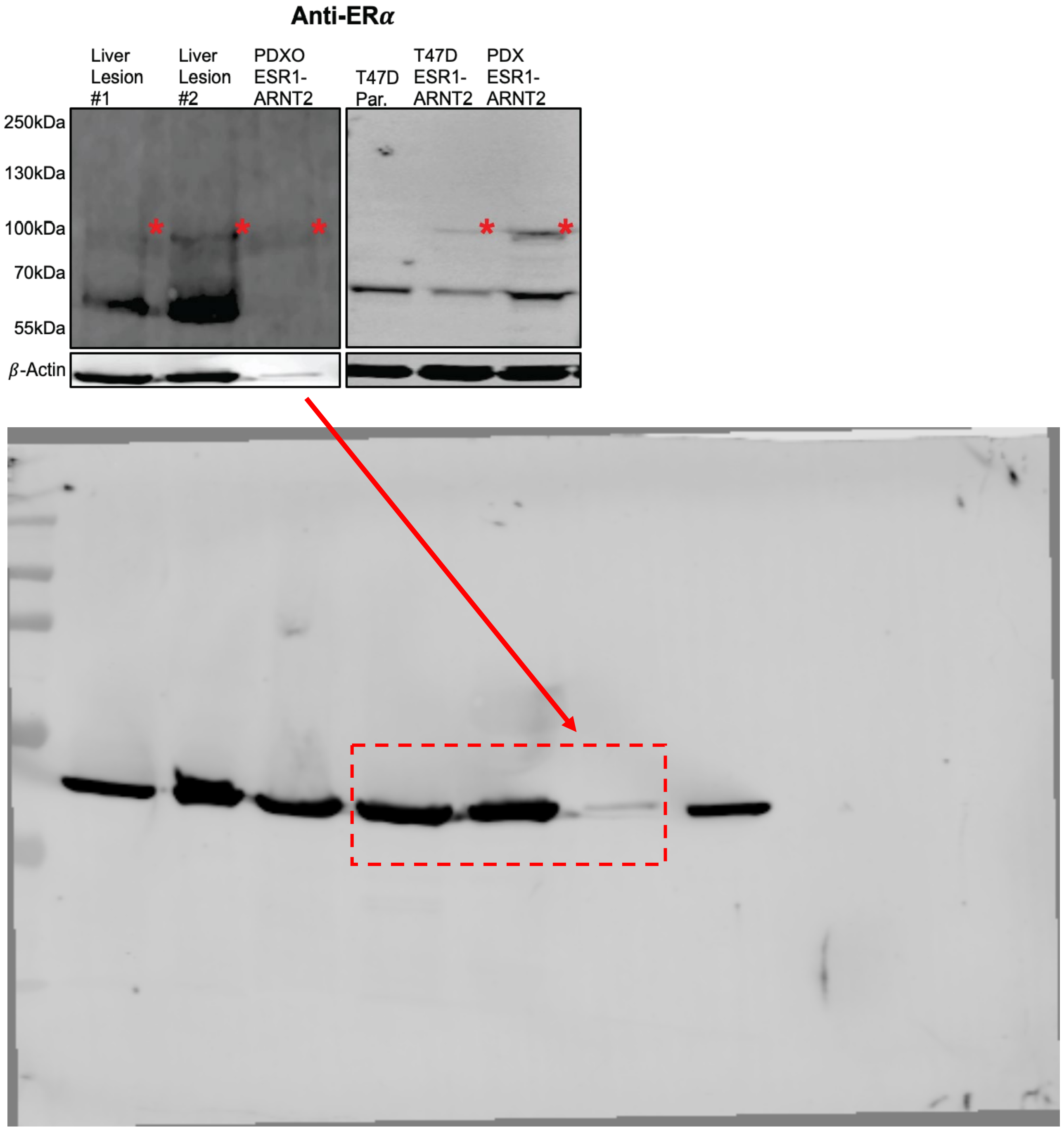

Anti-ERα
Liver Lesion #1
Liver Lesion #2
PDXO ESR1-ARNT2
T47D Par.
T47D ESR1-ARNT2
PDX ESR1-ARNT2
250kDa
130kDa
100kDa
70kDa
55kDa
β-Actin

Figure 8D

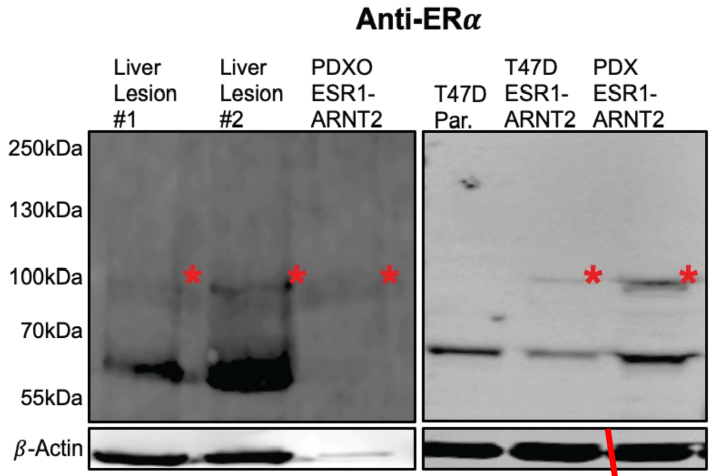

Anti-ERα
Liver Lesion #1
Liver Lesion #2
PDXO ESR1-ARNT2
T47D Par.
T47D ESR1-ARNT2
PDX ESR1-ARNT2
250kDa
130kDa
100kDa
70kDa
55kDa
β-Actin

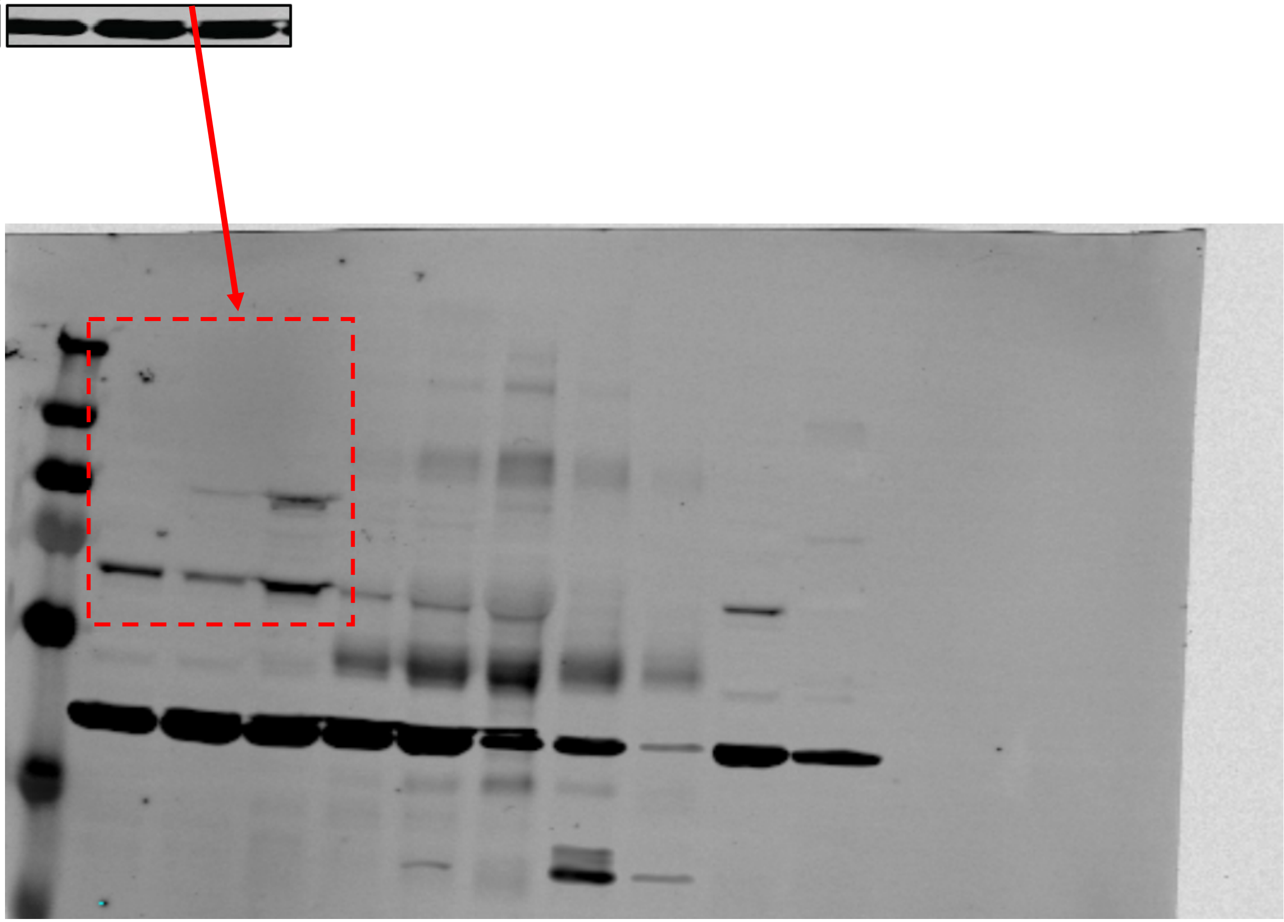

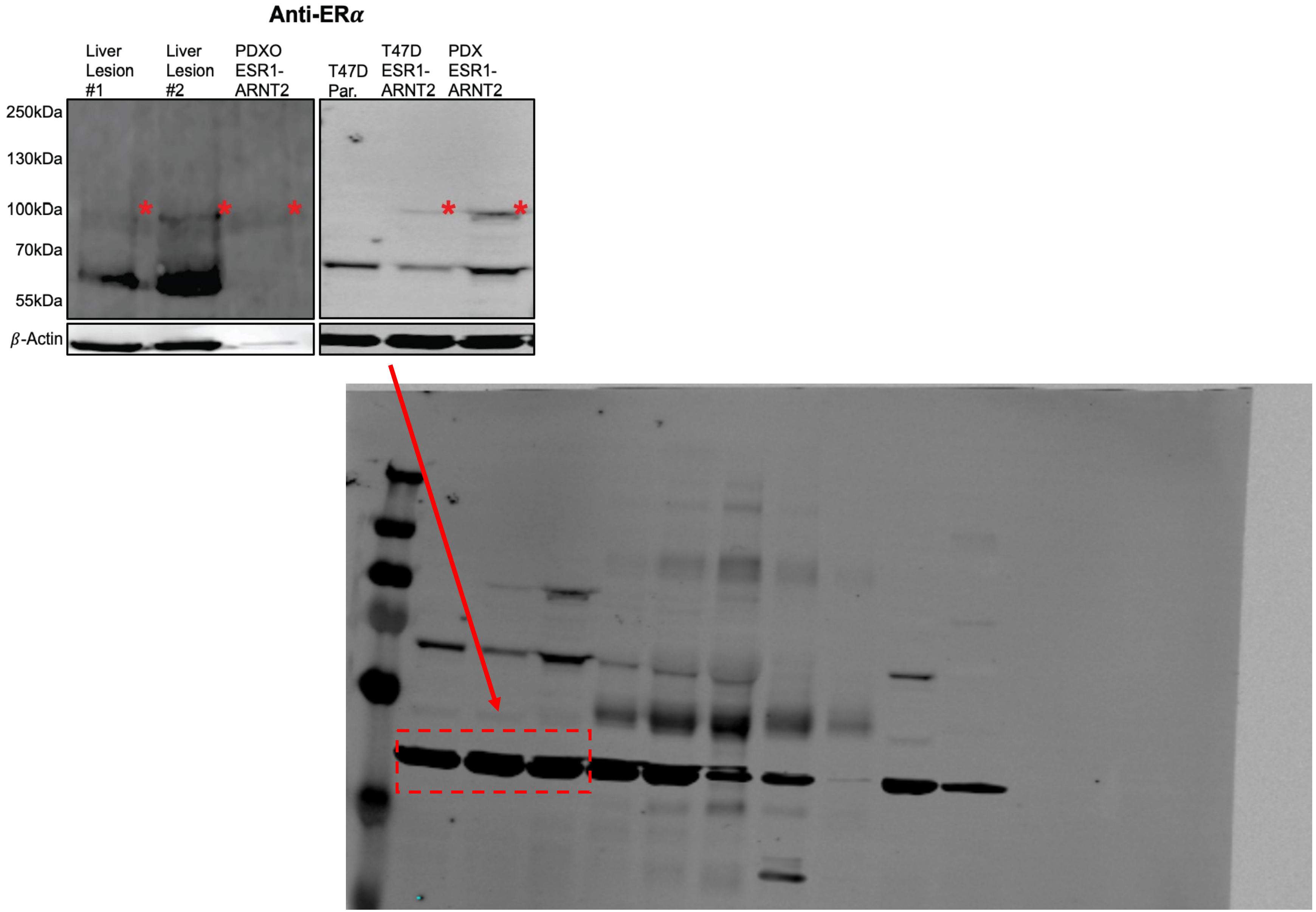
Anti-ERα
Liver Lesion #1
Liver Lesion #2
PDXO ESR1-ARNT2
T47D Par.
T47D ESR1-ARNT2
PDX ESR1-ARNT2
250kDa
130kDa
100kDa
70kDa
55kDa
β-Actin

Figure 8D
