# Supplementary material for: Hope for OTHERS (Our Tissue Helping Enhance Research & Science): research results from the University of Pittsburgh rapid autopsy program for breast cancer
Source: Breast Cancer Res. 2025 Jun 19;27:111. doi: 10.1186/s13058-025-02014-9 (PMC12180227; doi:10.1186/s13058-025-02014-9)
Supplement: Supplementary file 3 — Supplementary material 3 (Supplementary Figures). [file 13058_2025_2014_MOESM3_ESM.docx]

**Figure S1: Histogram for time from consent to death per patient.**


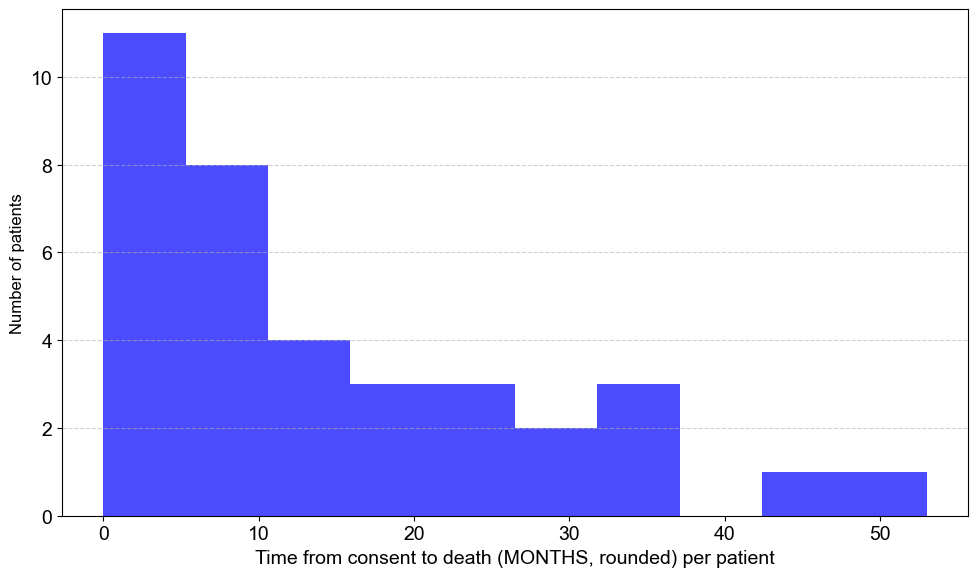


**Figure S1**: Histogram summarizing our time from consent to death in months, rounded.

**Figure S2: Pathway scores comparison between fixed and frozen tissue.**


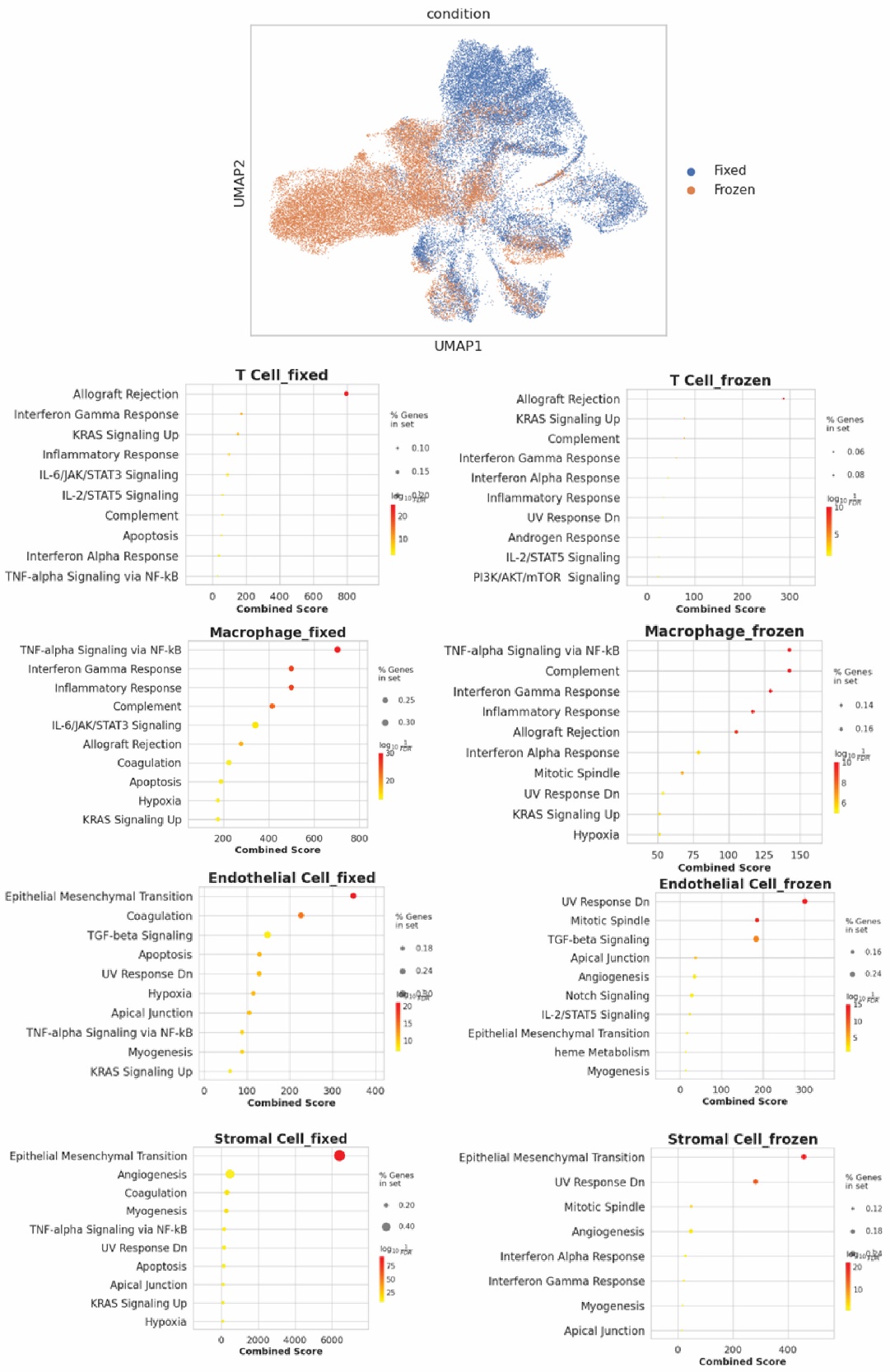


Figure S2. Downstream single cell analysis showing that fixed sequencing has better gene set percentage overlap and higher scores on average.

**Figure S3: Illustration of difference between chunked and minced tissue.**


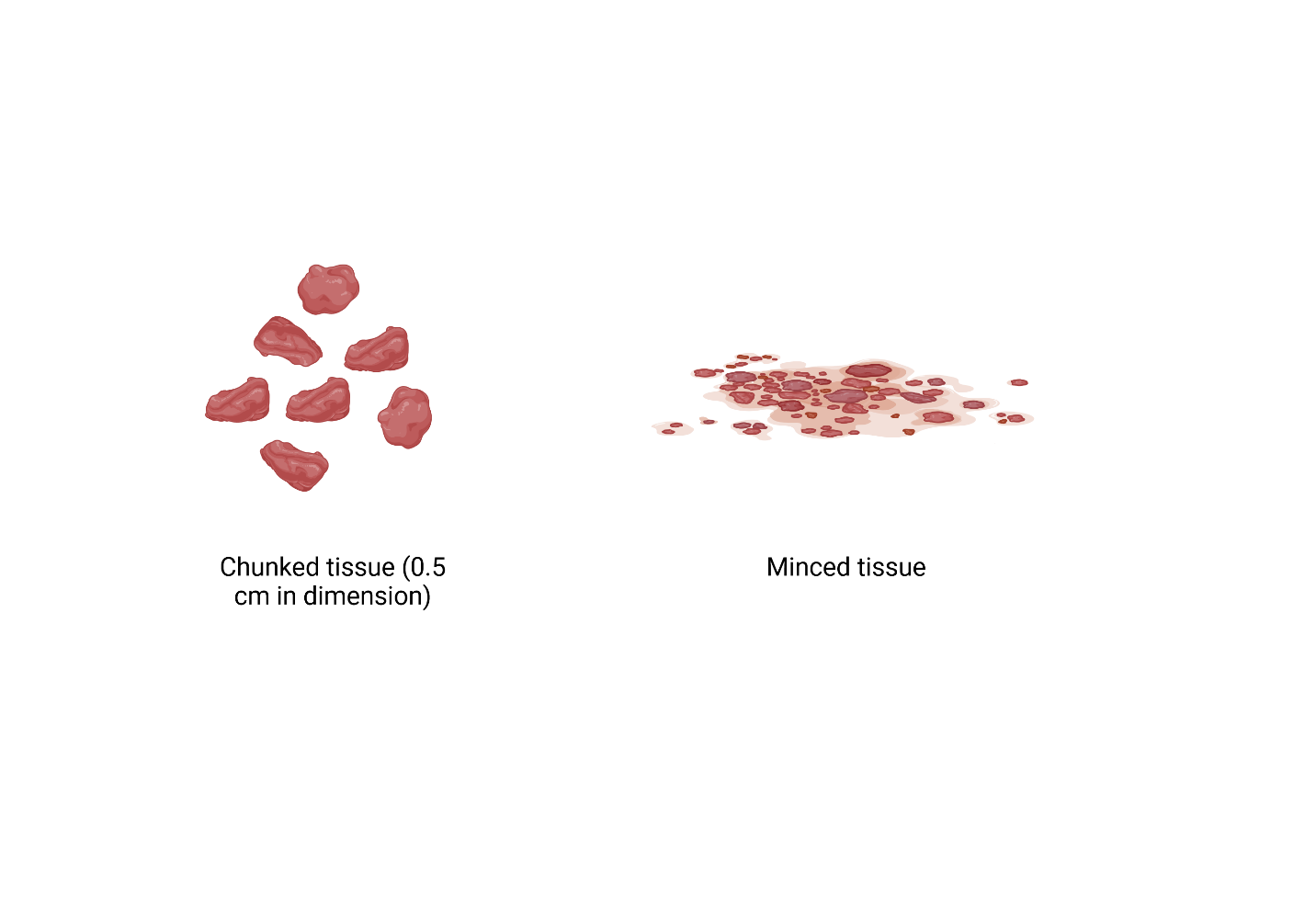


Figure S3. Illustration of the difference between chunked and minced tissue. Chunk tissues are preserved in 0.5cm – 1cm chunks. Minced tissues are chopped finely with a single-edged razor blade from an adjacent site.
